# Supplementary material for: High-Fat Diets with Differential Fatty Acids Induce Obesity and Perturb Gut Microbiota in Honey Bee
Source: Int J Mol Sci. 2021 Jan 15;22(2):834. doi: 10.3390/ijms22020834 (PMC7830725; doi:10.3390/ijms22020834)
Supplement: Supplementary file 1 [file ijms-22-00834-s001.pdf]

Supplementary Materials: Supplementary Table 1, Supplementary Figure 1 and 2.

Supplementary Table 1. Diet nutrient and energy composition in each diet.

	NDS		NDP		HFDS		HFDP	
	g %	kcal %	g %	kcal %	g %	kcal %	g %	kcal %
**Protein**	4	4	4	4	4	3	4	3
**Carbohydrate**	94	92	94	93	88	80	88	80
**Fat**	2	4	2	4	8	17	8	16
**kcal/g**	4.1		4.1		4.4		4.4	
**Ingredient**	g	kcal	g	kcal	g	kcal	g	kcal
**Casein**	2	8	2	8	2	8	2	8
**Sucrose**	50	200	50	200	50	200	50	200
**Soybean oil**	0.918	8.262			4.59	41.31		
**Palm oil**			0.912	8.208			4.56	41.04
**Total**	52.918	216.262	52.912	216.208	56.59	249.31	56.56	249.04

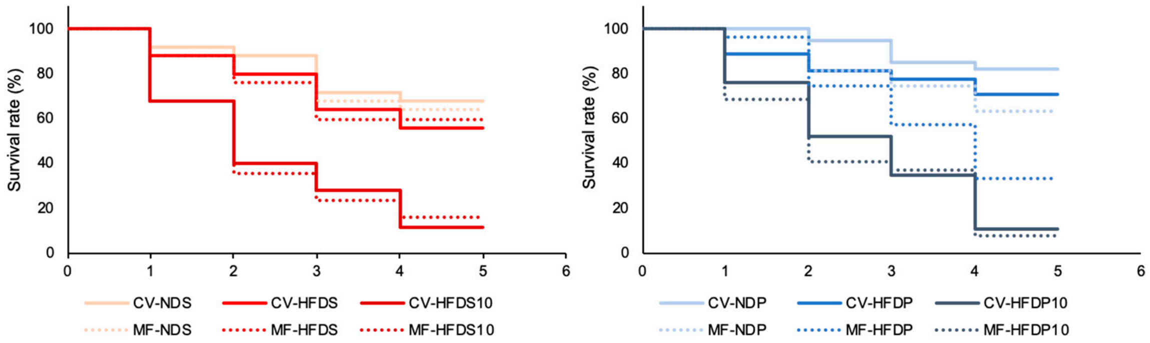

Supplementary Figure 1. Survival rates of honey bees in preliminary experiments. Dashed and solid lines correspond to MF and CV bees. Each line represents the average value of bee individuals (n = 25) from three cup cages. ND, HFD and HFD10 represent for fed with normal dietary fat (1%), high fat diet (5% fat) and extra high fat diet (10% fat). S and P stands for soybean oil and palm oil. Day 0 is the first day feeding dietary fats to bees.

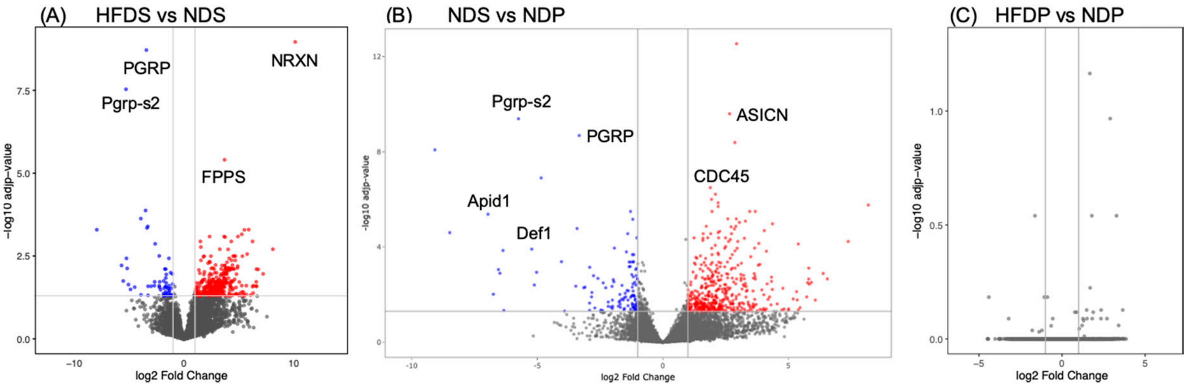

Supplementary Figure 2. Volcano plots of DEGs between (A) HFDS and NDS, (B) NDS and NDP, (C) HFDP and NDP (from left to right).
